# NK Receptors Replace CD28 As the Dominant Source of Signal 2 for Cognate Recognition of Cancer Cells by TAA-specific Effector CD8^+^ T Cells

**DOI:** 10.21203/rs.3.rs-3399211/v1

**Published:** 2023-10-19

**Authors:** Bowen Dong, Nataša Obermajer, Takemasa Tsuji, Junko Matsuzaki, Cindy Bonura, Henry Withers, Mark Long, Colin Chavel, Scott H. Olejniczak, Hans Minderman, Robert P. Edwards, Walter J. Storkus, Pedro Romero, Pawel Kalinski

**Affiliations:** 1 Department of Immunology, Roswell Park Comprehensive Cancer Center, Buffalo, NY, United States; 2 Department of Medicine, Roswell Park Comprehensive Cancer Center, Buffalo, NY, United States; 3 Department of Biostatistics and Bioinformatics, Roswell Park Comprehensive Cancer Center, Buffalo, NY, United States; 4 Flow and Immune Analysis Shared Resource, Roswell Park Comprehensive Cancer Center, Buffalo, NY, United States; 5 Department of Surgery, University of Pittsburgh School of Medicine, Pittsburgh, PA, United States; 6 Department of OB-GYN, University of Pittsburgh School of Medicine, Pittsburgh, PA, United States; 7 Department of Dermatology , University of Pittsburgh School of Medicine, Pittsburgh, PA, United States; 8 Department of Immunology, University of Pittsburgh School of Medicine, Pittsburgh, PA, United States; 9 University of Lausanne and Ludwig Institute for Cancer Research, Lausanne, Switzerland.

## Abstract

CD28-driven “signal 2” is critical for naïve CD8^+^ T cell responses to dendritic cell (DC)-presented weak antigens, including non-mutated tumor-associated antigens (TAAs). However, it is unclear how DC-primed cytotoxic T lymphocytes (CTLs) respond to the same TAAs presented by cancer cells which lack CD28 ligands. Here, we show that NK receptors (NKRs) DNAM-1 and NKG2D replace CD28 during CTL re-activation by cancer cells presenting low levels of MHC I/TAA complexes, leading to enhanced proximal TCR signaling, immune synapse formation, CTL polyfunctionality, release of cytolytic granules and antigen-specific cancer cell killing. Double-transduction of T cells with recombinant TCR and NKR constructs or upregulation of NKR-ligand expression on cancer cells by chemotherapy enabled effective recognition and killing of poorly immunogenic tumor cells by CTLs. Operational synergy between TCR and NKRs in CTL recognition explains the ability of cancer-expressed self-antigens to serve as tumor rejection antigens, helping to develop more effective therapies.

## Introduction

The “two-signal” paradigm of T cell activation, developed in 1970 [1] and refined over the past 50 years, explains the “self/non-self” discrimination by adaptive immune cells. Resting T cells typically avoid responses against TCR-binding antigens (signal 1) expressed by healthy cells which do not provide costimulatory signal 2, while selectively respond to signal 1 provided jointly with signal 2 on (pathogen-) activated antigen-presenting cells (APCs) [2]. The CD28-mediated costimulatory signal 2 is critical for initial priming of naïve T cells. Upon binding to its cognate ligands B7.1 (CD80) and B7.2 (CD86) expressed by activated dendritic cells (DCs), CD28 mediates multiple costimulatory effects, reducing the threshold for cognate TCR triggering, promoting T cell cytokine production, survival and clonal expansion [2, 3].

In contrast to the strict requirement for CD28-mediated costimulation during the (cross)priming of naïve CD8^+^ T cells, the role of signal 2 in the reactivation of effector CD8^+^ T cells (cytotoxic T lymphocytes; CTLs) is less clear, especially within the context of anti-cancer responses. Expression of “classical signal 2” ligands is restricted to professional APCs, with non-hematologic (solid) cancers failing to express these molecules. Meanwhile, activated CTLs upregulate expression of CTLA-4, a much higher affinity receptor for B7.1/B7.2, with an inhibitory function [2]. Moreover, despite the conceptual specter of autoimmune pathology, CTLs can target cancer-overexpressed non-mutated tumor-associated antigens (TAAs) without damaging healthy tissues expressing the same antigens, although typically at lower levels. This raises the question of whether effector CTL receive autonomous TCR-mediated signals in a signal 2-independent fashion (intrinsically enhanced functional avidity) or via alternative (non-CD28) costimulatory pathways in order to recognize and kill the B7.1/B7.2-negative TAA-expressing cancer cells. Inclusion of integral CD28-signaling elements in second-generation chimeric antigen receptor (CAR) constructs has proven effective in promoting the survival and antitumor efficacy of second-generation CAR-T cells [4], supporting the operational advantages of signal 2 provided during the effector phase of anti-cancer responses.

DNAM-1 and NKG2D are activating NK receptors (NKRs) expressed by human NK cells and CD8^+^ T cells. Unlike CD28 ligands which are expressed mainly by professional APCs, the ligands for NKRs are also expressed by stressed, infected and mutated cells [5, 6]. While NKRs have been shown to be important for CTL function, the mechanism of their involvement in anti-cancer responses remains elusive. Human *ex vivo* studies involving CTL clones generated in the presence of high-dose cytokines, such as IL-2 and IL-15 have indicated that DNAM-1 and NKG2D can either costimulate TCR-driven activation or induce TCR-independent activation of CD8^+^ T cells [7–13], raising a possibility that these NKRs may contribute to CD28-independent (re)activation of DC-primed CTLs responding to TAAs presented by distressed cancer cells.

Guided by these considerations, we evaluated the roles of DNAM-1 and NKG2D as components of “alternative signal 2” at different stages of DC-induced TAA-specific CTL activation. We observed that human CTLs (cross)primed by DCs or by DC-mimicking stimuli switch from CD28 to NKRs as dominant costimulatory receptors, enabling effective TCR-mediated recognition of cancer cells expressing low levels of MHC I/TAA peptide complexes. We found that DNAM-1, and to a lesser extent NKG2D, facilitate CTL activation by weak TCR stimuli, resulting in enhanced CTL polyfunctionality and effector function. Analysis of TCGA data revealed that DNAM-1 and NKG2D expression is strongly associated with intratumoral CD8^+^ T cells (rather than NK cells), being critical for the long-term survival in melanoma patients. Accordingly, enhanced delivery of such NKR-dependent alternative signal 2 as a result of NKR overexpression in TCR-transgenic T cells or by chemotherapy-driven elevation of NKR ligands on cancer cells, allowed effective recognition and killing of weakly immunogenic cancer cells, demonstrating the potential for targeting this pathway in adoptive T cell therapies and other forms of immunotherapy.

## Results

### Naïve and effector CD8^+^ T cells rely on, respectively, CD28 and DNAM-1, as dominant costimulatory molecules

CD28 is constitutively expressed on naïve CD8^+^ T cells but downregulated on activated CD8^+^ T cells, with the expression of its ligands B7.1 and B7.2 typically missing from non-hematologic cells, including most solid tumor cells [2]. In contrast, many activating NKRs, including DNAM-1 and NKG2D, are upregulated following CD8^+^ T cell activation (**Extended Data Fig. 1A, B**). Moreover, ligands of DNAM-1 (PVR and Nectin2) and NKG2D (MICA, MICB and ULBP1–6) are commonly expressed on human cancer cell lines of diverse tissue histology, such as colon cancer (SW620), ovarian cancer (OVCAR3) or melanoma (2183-Her4, Mel526, Mel624) (**Extended Data Fig. 1C,** *note that other NKR-Ls were expressed either in a cell-line-specific manner or at much lower levels*).

**Figure 1.**
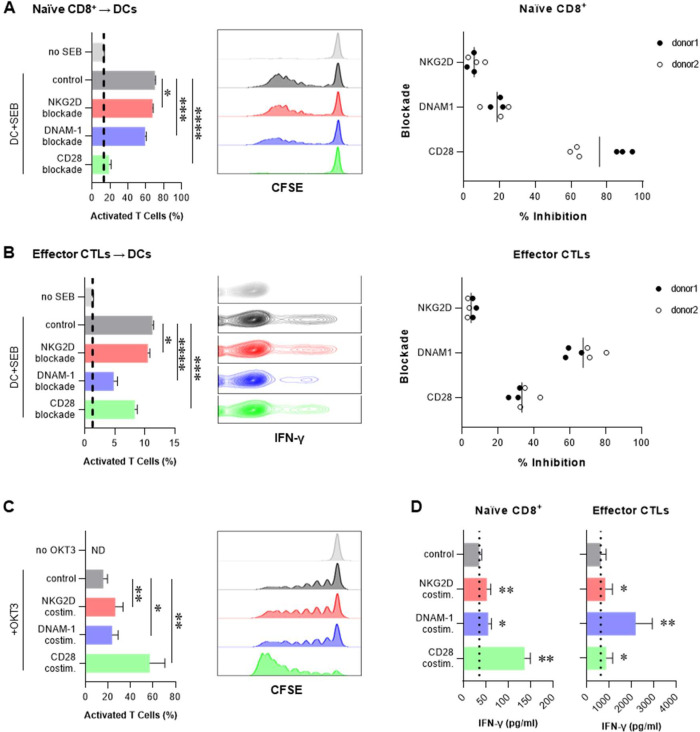
Naïve and effector CD8^+^ T cells rely on the CD28 versus DNAM-1 as the dominant costimulatory pathways. **(A)** Polyclonal activation of naïve CD8^+^ T cells by SEB-pulsed DCs in the presence of blocking antibodies against indicated receptors. *Left:* Percentages of CFSE^low^ proliferating cells (mean ± SD of triplicate cultures). *Center:* Representative flow cytometry histograms of T cell CFSE dilution. *Right:* Summary data of triplicate cultures from two donors showing inhibitory effect of each blocker on naïve cell activation, calculated as: % inhibition = [% Activated T cells (control) - % Activated T cells (blockade)] / [% Activated T cells (control) - % Activated T cells (no SEB)] × 100. (**B**) Re-activation of Dynabead-induced CTLs by SEB-pulsed DCs in the presence of blocking antibodies against indicated receptors. *Left:* Percentages of IFN-γ^+^ CTLs (mean ± SD of triplicate cultures). *Center:* Representative contour plots of side scatter and IFN-γ expression. *Right:* Summary data of triplicate cultures from two donors showing inhibitory effect of each blocker on CTL re-activation. (**C**) Activation of naïve CD8^+^ T cells by immobilized αCD3 (OKT3) with different costimulations. *Left:* Percentages of CFSE^low^ proliferating cells (n=3 donors; mean ± SEM). *Right:* Histograms showing CFSE dilution of naïve CD8^+^ T cells activated by immobilized OKT3 with different costimulatory signals in a representative donor. (**D**) Roles of CD28 versus NKR costimulation in naïve CD8^+^ T cells versus CTLs following stimulation with immobilized αCD3 (OKT3). Histograms showing IFN-γ secretion by naïve CD8^+^ T cells (*left*) or Dynabead-induced CTLs (*right*) activated by immobilized OKT3 with the indicated costimulatory signals (n=3 donors; mean ± SEM). Data were analyzed by two-tailed unpaired t test (**A** and **B**), or two-tailed ratio paired t test (**C** and **D**). *P < 0.05, **P < 0.01, ***P < 0.001, ****P < 0.0001. SEB: Staphylococcus Enterotoxin B.

To test the contribution of CD28 versus NKRs in the activation of naïve and effector CD8^+^ T cells, we cocultured naïve CD8^+^ T cells or Dynabead-induced CTLs with SEB-pulsed DCs and compared the inhibitory effects of blocking NKG2D, DNAM-1, or CD28 on resultant T cell activation. Blockade of CD28 engagement by CTLA4-Ig strongly inhibited the proliferation of naïve CD8^+^ T cells by DCs, while blockade of either NKG2D or DNAM-1 showed only weak inhibitory effects (Fig. 1A). In striking contrast, during the interaction of preactivated CTLs with DCs, DNAM-1 but not CD28 blockade showed a dominant inhibitory effect on IFN-γ production (Fig. 1B). These observations were confirmed using an alternative approach where naïve CD8^+^ T cells or Dynabead-induced CTLs were stimulated by immobilized anti-CD3 (OKT3) either alone or in combination with agonist antibodies reactive with NKG2D, DNAM-1 or CD28 (*note that neither naïve nor effector cells were activated in the absence of the OKT3 antibody*). Consistently, CD28-costimulation was key to the activation of naïve CD8^+^ T cells, based on both proliferation (Fig. 1C) and IFN-γ secretion (Fig. 1D left) as functional readouts. However, in case of CTL (re)activation, it was the DNAM-1 signaling which proved dominant in costimulating T cell secretion of IFN-γ, with NKG2D and CD28 signaling demonstrating only modest costimulatory effects (Fig. 1D right). These data show that CD8^+^ T cells at different stages of activation preferentially benefit from divergent costimulatory pathways and that CTLs acquire the capacity to use DNAM-1 and NKG2D to receive an “alternative signal 2” in the absence of CD28 costimulation.

### Signals from DNAM-1 and NKG2D synergize with TCR to enable effective recognition of cancer cells presenting low-levels of MHC I/TAA peptide complexes

CD28 costimulation is known to amplify the TCR-driven signal 1, assisting (naïve) T cell activation under physiologic levels of TCR stimulation [3]. Therefore, we tested whether the relative impact of NKR-mediated costimulation also depends on the strength of TCR-pMHC-I-delivered signals, using MART-1 (Melan A), a melanocyte differentiation antigen expressed by melanoma and normal melanocytes, as a model TAA [14–16]. MART-1-specific CTLs were induced by *in vitro* sensitization (IVS) using autologous MART-1 peptide-loaded DCs and CD8^+^ T cells isolated from healthy normal HLA-A*02:01^+^ donors (**Extended Data Fig. 2A-C**). MART-1-negative (but HLA-A*02:01^+^) SW620 colorectal cancer cells were loaded with increasing concentrations of MART-1 peptide to evaluate the ability of DC-primed CTLs to recognize cancer cells presenting low- vs. high-levels of the TAA-derived peptide. Notably, despite the expression of multiple ligands for DNAM-1 and NKG2D by SW620 cells (**Extended Data Fig. 1C**), DC-sensitized CTLs exclusively recognized and killed only MART-1-loaded cancer cells, while fully ignored MART-1-negative cells. These results demonstrate that the activation of DC-sensitized CTLs is fully dependent on TCR-delivered signal 1, unlike LAK/CIK-type activation by NKR signals [9, 11] (**Extended Data Fig. 2D, E**).

**Figure 2.**
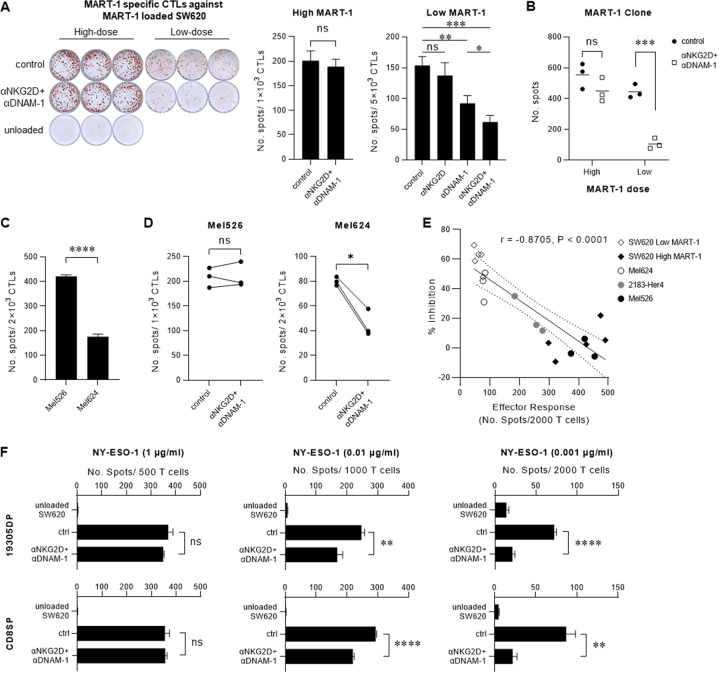
DNAM-1 and NKG2D facilitate the TCR-dependent recognition of cancer cells expressing low-level TAAs. In **A** and **B**, MART-1-negative SW620 cells were loaded with high concentration (1 μg/ml) or low concentration (0.01 μg/ml) of MART-1 peptide, as a model of cancer cells expressing high or low levels of TAA. NKG2D and DNAM-1 were blocked by antibodies. (**A**) IFN-γ secretion by DC-sensitized MART-1-specific CTLs against MART-1-loaded cancer cells in the absence or presence of the indicated blockers. Data show a representative image of ELISPOT performed in triplicate wells per condition (*left*) and quantification of spots (*right:* n=5 donors; mean ± SEM). (**B**) Effect of NKG2D and DNAM-1 blockade on IFN-γ secretion by patient-derived MART-1-specific clone (clone 40) against MART-1-loaded cancer cells. Data show quantification of spot number from a representative experiment performed in triplicate cultures per condition. (**C**) Different activation levels of DC-sensitized MART-1-specific CTLs by Mel562 versus Mel624 melanoma cells. Data show quantification of spot number (mean ± SD of triplicate cultures). (**D**) IFN-γ secretion by DC-sensitized MART-1-specific CTLs against weakly versus strongly stimulatory melanoma cells in the absence or presence of NKR blockade. Data show quantification of spot number (n=3 donors, each pair of dots represents means of paired triplicate cultures from each individual donor). (**E**) Correlation between the inhibitory effect of NKG2D/DNAM-1 blockade and the strength of effector response in the recognition of cancer cells (melanoma cells or MART-1-loaded SW620) by DC-sensitized MART-1-specific CTLs. (**F**) IFN-γ secretion by NY-ESO-1 specific TCR-transduced CD8^+^ T cells (19305DP and CD8SP) against cancer cells loaded with decreasing doses of NY-ESO-1 peptides, with or without NKR blockade (triplicate cultures per condition; mean ± SD). Data were analyzed by two-tailed ratio paired t test (**A** and **D**), or two-tailed unpaired t test (**B, C** and **F**). Data in (**E**) were modeled by simple linear regression and analyzed by Pearson correlation. *P < 0.05, **P < 0.01, ***P < 0.001, ****P < 0.0001, not significant (ns): *P* > 0.05.

The impact of NKG2D and DNAM-1 blockade on CTL recognition of cancer cells presenting high- or low-dose MART-1 was determined by IFN-γ ELISpot. As shown in Fig. 2A, blockade of NKG2D and DNAM-1, either individually or in combination, had no detectable inhibitory effect on CTL recognition of SW620 cells loaded with high-dose MART-1 peptide. In contrast, blockade of DNAM-1 inhibited CTL recognition of SW620 cells pulsed with low-dose MART-1 peptide, with the maximal inhibition observed upon coordinate blockade of both DNAM-1 and NKG2D. As in the case of MART-1-specific CTLs induced from healthy donors, a MART-1-specific CTL clone isolated from tumor infiltrating lymphocytes from an HLA-A*02:01^+^ melanoma patient [17] also required NKG2D and DNAM-1 for optimal recognition of HLA-matched tumor cells loaded with low, but not high, doses of the MART-1 peptide (Fig. 2B).

### Requirement for NKR-mediated costimulation depends on the immunogenicity of cancer cells

In addition to heterogenous levels of TAA expression, downregulation of MHC I and cell adhesion molecules are also commonly targeted by tumor cells to escape immune surveillance [18–20]. Interestingly, while anti-MART-1 CTL recognition of cancer cells presenting high levels of antigen typically did not require participation of either NKG2D or DNAM-1 (Fig. 2A, B), partial blockade of either MHC-I or CD2/LFA-3 revealed a requirement for both NKRs in antigen-specific T cell recognition of high-dose MART-1 peptide-loaded cancer cells (**Extended Data Fig.** 3).

Consistent with these findings, blockade of NKRs inhibited CTL recognition of weakly-immunogenic Mel624 melanoma cells, but not the more immunogenic Mel526 melanoma cell line (Fig. 2C, D, please note the different levels of the overall reactivity to these two HLA-A*02:01^+^*, MART-1*^+^ *cell lines*). The key role of alternative signal 2 in the recognition of weakly-immunogenic cancer cells was further demonstrated by a negative correlation between the overall strength of effector response and the inhibitory effect of NKR blockade observed across our different experiments and models (Fig. 2E).

This same pattern was observed during the recognition of cancer cells loaded with increasing doses of NY-ESO-1 (a TAA shared by normal testis and multiple tumors) by NY-ESO-1-specific TCR-transduced CD8^+^ T cells (19305DP and CD8SP [21]): NKR blockade prevented the CTL recognition of low-TAA-presenting cancer cells with a progressively reduced role for T cell recognition of tumors expressing higher levels of MHC I/TAA peptide complexes (Fig. 2F).

### DNAM-1 and NKG2D enable CTL-mediated killing of tumor cells expressing low-levels of cognate antigen

**Figure 3.**
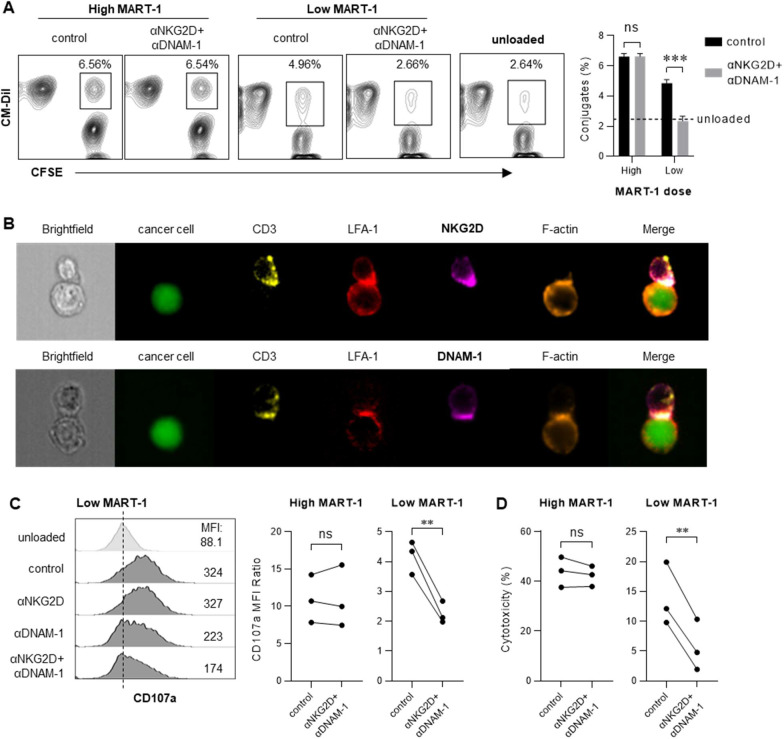
DNAM-1 and NKG2D assist CTL-mediated cytolysis against cancer cells expressing low-level TAAs. (**A**) Contour plots (*left*) and histogram (*right*) showing the percentages of CM-Dil^+^CFSE^+^ conjugates formed by CTLs and cancer cells loaded with different doses of MART-1 in the absence or presence of NKR blocking antibodies (triplicate cultures per condition; mean ± SD). (**B**) Representative brightfield pictures of CTL-cancer cell conjugates, and fluorescent pictures showing CFSE labelled cancer cells, CD3, LFA-1, NKG2D or DNAM-1, F-actin and their colocalization. (**C**) Degranulation of CTLs under indicated conditions was monitored by surface expression of CD107a on CTLs and presented as histograms with mean fluorescent index (MFI) (*left*) or the ratios of CD107a MFI in each of the indicated conditions to the MFI of unloaded control (*right*, n=3 donors; each pair of dots represents means of paired triplicate cultures from each individual donor). (**D**) Killing of target cells by DC-sensitized MART-1-specific CTLs under the indicated conditions was analyzed by LDH cytotoxicity assay (n=3 donors; each pair of dots represents means of paired triplicate cultures from each individual donor). Data were analyzed by two-tailed unpaired t test (**A**), or two-tailed ratio paired t test (**C** and **D**). **P < 0.01, ***P < 0.001, not significant (ns): *P* > 0.05.

We next tested the involvement of DNAM-1 and NKG2D in the sequential steps of CTL-mediated cytolysis, including conjugate formation, cytoplasmic rearrangement, and degranulation. Conjugate formation was detected by coculture of CM-Dil labelled MART-1-specific CTLs with CFSE labelled MART-1-loaded SW620. Blockade of NKG2D and DNAM-1 strongly inhibited the development of CTL conjugation with low-dose MART-1-loaded cancer cells, without affecting conjugation with high-dose MART-1-loaded cancer cells (Fig. 3A). ImageStream analyses revealed that both NKG2D and DNAM-1 were polarized at the CTL-cancer cell contact zone, and colocalized with markers of the immune synapse including CD3, LFA-1, and F-Actin (Fig. 3B). Consistently, CTL degranulation (measured as CD107a translocation to the T cell plasma membrane) in response to low-dose MART-1-loaded cancer cells was seen to be critically dependent on NKRs (Fig. 3C). Furthermore, LDH cytotoxicity assays confirmed that NKG2D and DNAM-1 blockade inhibited the T cell killing of low-dose (but not high-dose) MART-1-loaded cancer cells (Fig. 3D).

### DNAM-1 and NKG2D costimulation enhances the polyfunctionality of CTLs activated by low-level TCR triggering

**Figure 4.**
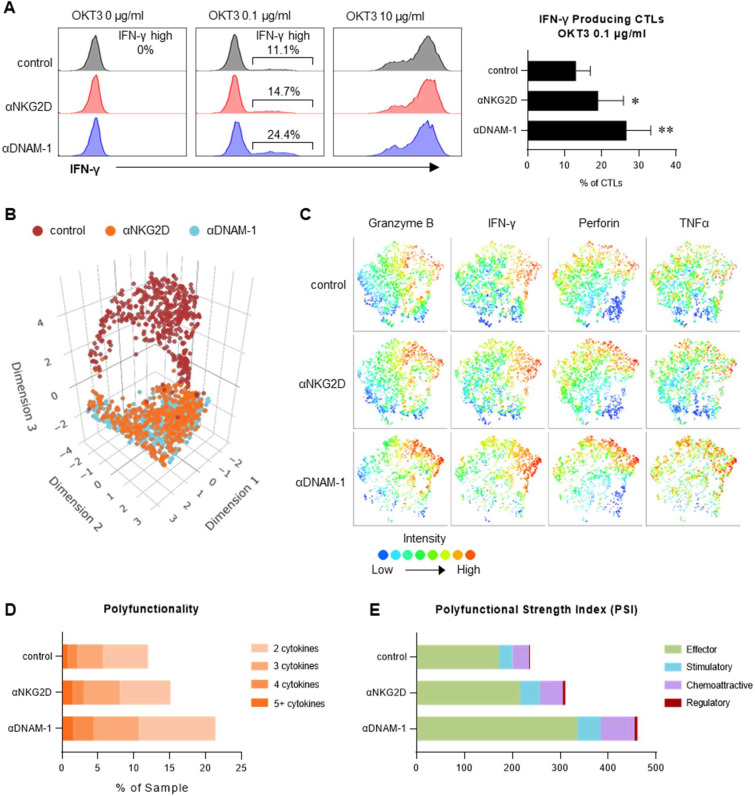
DNAM-1 and NKG2D costimulation enhances the polyfunctionality of CTLs activated by low-level TCR triggering. Dynabead-induced CTLs were activated by immobilized antibodies. (**A**) Intracellular IFN-γ levels in Dynabead-induced CTLs activated by increasing concentrations of OKT3 in the absence or presence of NKR costimulation. *Left:* Representative flow cytometry histograms showing intracellular IFN-γ levels of differentially stimulated CTLs. *Right:* Results from 3 donors, expressed as percentages of IFN-γ-producing CTLs activated by low-dose OKT3 in the absence or presence of NKR costimulation (n=3 donors; mean ± SEM). (**B** to **E**) Single-cell secretome of CTLs activated under the indicated conditions were analyzed using Adaptive Immune ISOCODE **chips** and isoLight system (IsoPlexis), showing summary (mean) results of 2 separate experiments using different donors. (**B**) 3D-UMAP projection showing clusters of CTLs activated by OKT3 alone and CTLs activated by OKT3 plus NKR-costimulation. (**C**) Expression of effector cytokines was overlayed on t-SNE projections, showing intensity of cytokines in CTLs activated by low-dose OKT3 either alone or in combination with the indicated NKR-costimulation. (**D**) Polyfunctionality was calculated as the percentages of activated CTLs secreting ≥ 2 types of proteins. (**E**) Polyfunctional Strength Index (PSI) was computed as the percentage of polyfunctional cells, multiplied by the sum of the mean fluorescence intensity of the proteins secreted by those cells. Proteins were grouped and color-coded based on their functions: Effector: Granzyme B, IFN-γ, Perforin, TNFα; Stimulatory: GM-CSF, IL-2, IL-12, IL-15; Chemo-attractive: CCL4, CCL5; Regulatory: IL4, IL10, sCD40L. Data in (**A**) were analyzed by two-tailed ratio paired t test. *P < 0.05, **P < 0.01.

To characterize the impact of NKR costimulation on CTL secretomes, we employed a DC-free model of CD3/CD28-(pre)activated CTLs, which showed a similar dependence on DNAM-1, and to a lesser extent NKG2D in response to low dose anti-CD3 reactivation (Fig. 4A). The 3D-UMAP projection of single-cell secretomes of 32 mediators of adaptive immunity showed profound differences between the control (CD3-only activated) CTLs and the NKG2D- or DNAM-1-costimulated CTLs (Fig. 4B). DNAM-1, and to a lesser extent NKG2D, enhanced T cell secretion of effector function-associated factors including Granzyme B, IFN-γ, Perforin, and TNFα (Fig. 4C), and increased the number of individual CTLs secreting multiple factors (Fig. 4D). The polyfunctional strength index (PSI) reflects the ability of a T cell to carry out multiple functions and has been shown to predict the efficacy of immune therapies [22–24]. As shown in Fig. 4E, NKR-mediated costimulation of CTLs increased the PSI score and the ability of CTLs to secrete cytokines across all functional categories.

### CD28-like canonical costimulatory effects of DNAM-1 assist early stages of TCR-signaling

NKG2D signaling in NK cells and T cells is known to involve DAP10 adaptor protein which uses a similar YXNM motif as CD28 to mediate signal transduction [25, 26]. However, the optimal responsiveness of CD8^+^ T cells to NKG2D activation requires IL-15 or high dose IL-2 to induce DAP10 expression [11, 12]. Consistently, our results showed only a weak NKG2D-mediated costimulatory effect on CD8^+^ T cells induced in absence of high dose IL-2 or IL-15. In contrast, we observed that DNAM-1 was dominant in assisting TCR-driven activation. In case of NK cells, DNAM-1 transduces signals through SLP76/VAV1-PLCγ2 pathway, leading to the activation of transcriptional factors AP-1, NFAT, and NF-κB [6, 27], known to be also involved in TCR signaling.

**Figure 5.**
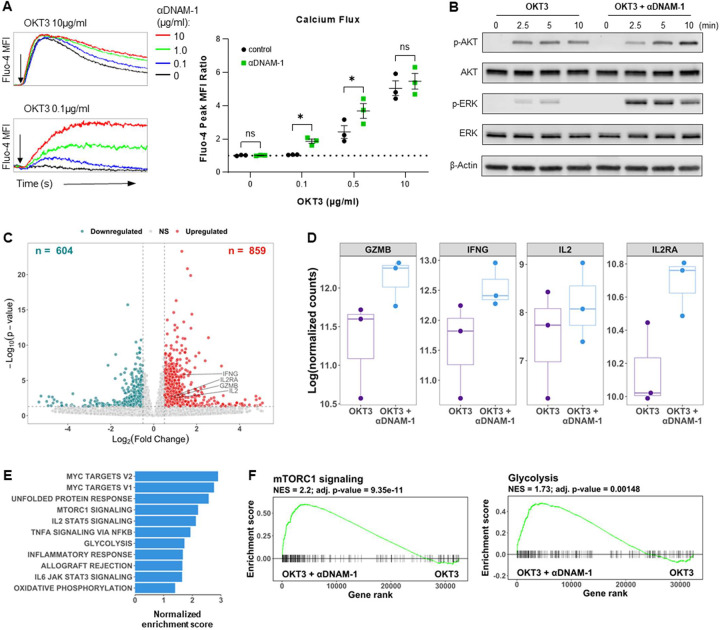
DNAM-1 costimulation lowers the threshold of TCR activation and assists proximal TCR signaling in effector CD8^+^ T cells. Dynabead-induced CTLs were activated by streptavidin crosslinking of biotinylated αCD3 (OKT3) with or without biotinylated αDNAM-1. (A) Induction of calcium flux by OKT3 with or without DNAM-1 costimulation. *Left:* Intracellular calcium levels over time in CTLs stimulated with OKT3 and different concentrations of αDNAM-1 (the arrow indicated the addition of streptavidin). *Right:* Results from 3 independent experiments were normalized by Fluo-4 Peak MFI Ratio calculated as Fluo-4 Peak MFI divided by baseline MFI (n=3 donors; mean ± SEM). (**B**) Western blot of phosphorylated AKT and ERK in CTLs stimulated with OKT3 or OKT3+αDNAM-1 at different timepoints. (**C-F**) RNA sequencing: Gene expression profiles of the OKT3-stimulated CTLs vs. OKT3+αDNAM-1-stimulated CTLs from 3 donors. (**C**) Volcano plot showing the differentially expressed genes (DEGs) in CTLs activated by OKT3 with or without DNAM-1 costimulation. DEGs with log2 fold change > 0.5 and adjusted *P* value< 0.05 were defined as significant. (**D**) Expression of GZMB, IFNG, IL2 and IL2RA in OKT3+αDNAM-1-stimulated CTLs compared to OKT3-stimulated CTLs. (**E**) Top enriched pathways in OKT3+αDNAM-1-stimulated CTLs compared to OKT3-stimulated CTLs from Gene Set Enrichment Analysis (GSEA). (**F**) Enrichment of mTORC1 signaling and glycolysis in OKT3+αDNAM-1-stimulated CTLs compared to OKT3-stimulated CTLs. Data in (**A**) were analyzed by multiple paired t test. *P < 0.05, not significant (ns): *P* > 0.05.

Since DNAM-1 enhanced the responsiveness of CTLs to weak TCR stimulation, we next tested whether DNAM-1 engagement decreases the threshold of TCR triggering. Dynabeads-induced CTLs were labelled with calcium indicator Fluo-4 and pre-incubated with different doses of biotinylated agonist anti-CD3 and anti-DNAM-1 antibodies. TCR and DNAM-1 signals were then triggered by cross-linking antibodies with streptavidin. We observed that activation of DNAM-1 increased the TCR-triggered intracellular Ca^2+^ in an αDNAM-1 dose-dependent manner. Moreover, DNAM-1 engagement allowed CTLs to respond to low-level CD3 stimulation which was insufficient to induce calcium flux by itself. In contrast, calcium flux in CTL induced by high-level CD3 engagement was minimally affected by DNAM-1 engagement (Fig. 5A). In the absence of TCR stimulation, even maximal DNAM-1 cross-linking induced only a low level of delayed calcium signaling in T cells (**Extended Data Fig. 4A**). We also observed that DNAM-1 was superior to CD28 in supporting TCR-triggered calcium flux in effector CTLs (**Extended Data Fig.** 4**B**). These results support the notion that DNAM-1 is dominant over CD28 for the costimulation of CD8^+^ T cell effector responses under conditions of weak TCR engagement. In addition to calcium flux induction indicative of calcineurin-NFAT pathway activation, we observed a robust increase of phosphorylated AKT and ERK in DNAM-1-costimulated CTLs which relates to the activation of the AKT/mTOR and Ras/MAPK pathways, respectively (Fig. 5B).

Using RNA sequencing, we compared gene expression profiles of CTLs (re)activated in the absence or presence of DNAM-1 costimulation. We observed 859 upregulated genes in DNAM-1-costimulated CTLs (log2FC > 0.5, p adj. < 0.05), including *IFNG, GZMB, IL2RA*, and *IL2* (Fig. 5C, D), key biomarkers of initial effector T cell responses [28]. Gene set enrichment analyses showed strong upregulation of TCR downstream transcriptional factors, including those involved in Myc pathway, IL2-STAT5 pathway and TNFα signaling, as well as the genes known to be regulated by CD28 costimulation such as the AKT-mTORc1 and glycolytic pathways (Fig. 5E, F). These results demonstrate that DNAM-1 delivers CD28-like canonical costimulatory effects, assisting early stages of TCR-driven (re)activation of CD8^+^ T effector cells.

### Enhanced NKR-mediated “alternative signal 2” allows killing of low-TAA-expressing cancer cells by human TCR-transgenic CD8^+^ T cells and DC-sensitized CTLs

**Figure 6.**
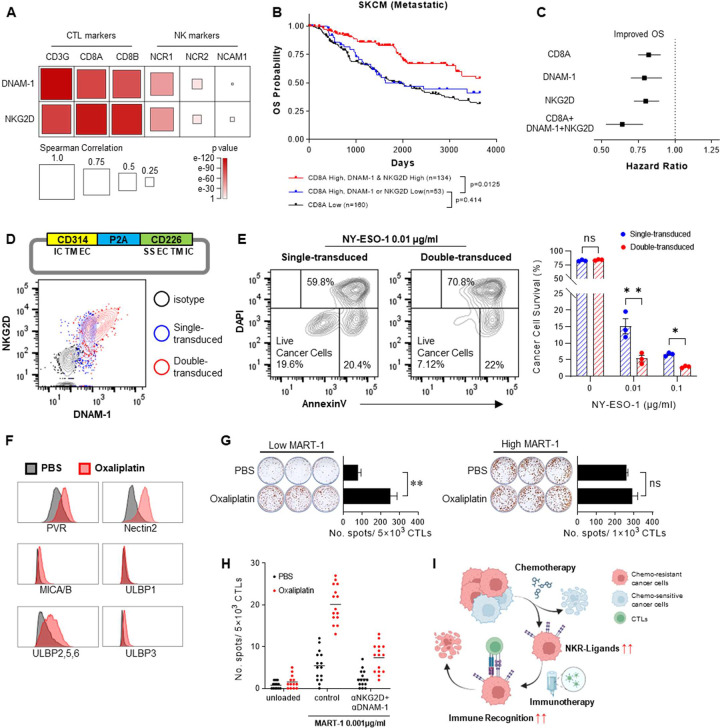
Overexpression of NKRs on CTLs or chemotherapy-induced elevation of NKR-Ls on cancer cells enables recognition and killing of low-TAA-expressing cancer cells. (**A-C**) Analysis of TCGA data from patients with metastatic melanoma (SKCM; n=360). (**A**) Correlation matrix showing the correlation between DNAM-1/NKG2D gene expression and CTL/NK cell lineage markers. Size and color of the squares represent Spearman correlation coefficient and p values, respectively (*see bottom of the panel for the values*). (**B**) Kaplan-Meier survival curves showing the 10-year overall survival probability of metastatic melanoma (SKCM) patients with different expression of *CD8A, DNAM-1*, and *NKG2D*. Data were analyzed by log-rank test. (**C**) Forest plot showing hazard ratios of *CD8A, DNAM-1, NKG2D* and their combination to estimate overall survival. (**D, E**) Enhanced tumor killing function of TCR/NKRs double-transduced CD8^+^ T cells. (**D**) Overexpression of NKG2D and DNAM-1 on TCR transduced CD8^+^ T cells. *Upper:* Design of the NKG2D/DNAM-1 co-expressing vector (IC: intracellular domain; TM: transmembrane domain; EC: extracellular domain; SS: spacer sequence). *Lower:* Contour plots comparing the expression of NKG2D and DNAM-1 on blood-isolated CD8^+^ T cells transduced with the TCR construct (single-transduced) or with the TCR construct and the additional NKG2D/DNAM-1 co-expressing vector (double-transduced). (**E**) Survival of SW620 cell loaded with increasing concentrations of NY-ESO-1 (0, 0.01, and 0.1 μg/ml) after 24-hour co-culture with single-transduced T cells or double-transduced T cells. Data show representative contour plots of AnnexinV and DAPI (*left*) and summary results of 3 independent experiments quantifying the percentage of AnnexinV^−^ DAPI^−^ surviving cancer cells (*right*, n=3 donors; mean ± SEM). (**F-I**) Enhanced delivery of NKR-mediated signal 2 allows for enhanced CTL recognition of chemotherapy-stressed cancer cells. (**F**) Comparison of expression of NKG2D and DNAM-1 ligands on untreated or oxaliplatin-treated (100 μM; 72 hours) SW620 cells. (**G**) IFN-γ secretion by DC-sensitized MART-1-specific CTLs against untreated or oxaliplatin-treated SW620 cells loaded with MART-1 peptide (low-dose: 0.01 μg/ml, high-dose: 1 μg/ml). Data show a representative image of ELISPOT performed in triplicate wells per condition and quantification of spot number from the same experiment (mean ± SD). (**H**) IFN-γ secretion by DC-sensitized MART-1-specific CTLs against SW620 untreated or pre-treated with oxaliplatin for 72 hours and loaded with 0.001 μg/ml MART-1 peptide. Data show summary result of 5 independent experiments performed in triplicate wells per condition. (**I**) Schematic depiction of sensitizing chemo-resistant cancer cells to immune recognition through upregulation of NKR ligands. Data in were analyzed by multiple paired t test (**E**), or two-tailed unpaired t test (**G**). *P < 0.05, **P < 0.01, not significant (ns): *P* > 0.05.

TCGA analysis showed that DNAM-1 and NKG2D expression is associated with improved overall survival in metastatic melanoma patients and correlated with genes of effector function, including *IFNG, GZMK*, and *GZMB* (**Extended Data Fig. 5A, B**). Unexpectedly, both DNAM-1 and NKG2D were most strongly correlated with intratumoral CTL markers *CD3G, CD8A*, and *CD8B*, rather than NK cell markers (Fig. 6A). Similar correlations were also observed in colon and ovarian cancer patients (**Extended Data Fig. 5C**). Strikingly, only the patients with both elevated *CD8A* and elevated *DNAM-1* and *NKG2D* showed superior survival to *CD8A*-low patients, while the patients with high *CD8A* expression but low expression of *DNAM-1* or *NKG2D* showed no survival advantage compared to patients with low *CD8A* expression, and significantly worse survival than patients with high expression of *CD8A, DNAM-1*, and *NKG2D*, suggesting that CD8^+^ T cells which do not express DNAM-1 and NKG2D are not effective in tumor control (Fig. 6B). Analysis of hazard ratios further indicated that combined expression of CD8A, DNAM-1, and NKG2D predicted prognosis better than each single gene expression (Fig. 6C).

Guided by these observations, we evaluated the relevance of our findings to cancer therapy and tested if the modulation of the levels of NKG2D and DNAM-1 on CTLs can be used to enhance their anti-tumor activity. We employed retroviral vectors encoding NKG2D/DNAM-1 to engineer overexpression of these NKRs by HLA-A*02:01-restricted NY-ESO-1 TCR-transgenic CD8^+^ T cells (Fig. 6D). As shown in Fig. 6E, the TCR/NKR “double-transduced” T cells demonstrated strongly elevated cytotoxic activity against peptide-loaded SW620 tumor cells when compared to the TCR-only “single-transduced” T cells, especially against low-TAA-expressing targets. Importantly, the “double-transduced” T cells did not show any increase in nonspecific killing of NY-ESO-1-unloaded cancer cells. These results demonstrate the potential for manipulating the T cell NKR levels to enhance the effectiveness of T cell recognition and killing of weakly immunogenic cancer cells.

Since DNA damage response is known to result in elevated expression of NKG2D and DNAM-1 ligands [5, 6, 29], we tested if chemotherapeutic agents can upregulate theses ligands and thus facilitate cancer cell recognition by DC-sensitized TAA-specific CTLs. Oxaliplatin and cisplatin, frequently used to treat colorectal and ovarian cancers, enhanced the expression of NKR ligands (NKR-Ls) on SW620 (colorectal) and SKOV3 (ovarian) cells, but not Caco-2 (colorectal). Notably, the upregulation of NKR-Ls was achieved with low-dose oxaliplatin and cisplatin which were insufficient for direct cytotoxic effects (**Extended Data Fig. 6**). Moreover, SW620 cells surviving prolonged high-dose oxaliplatin exposure exhibited elevated NKR-L expression (Fig. 6F). Oxaliplatin-treated SW620 triggered a significantly stronger response from MART-1-specific CTLs, compared to untreated SW620, especially when loaded with low-dose of MART-1 peptide (Fig. 6G). NKR blockade counteracted the enhanced CTL recognition of oxaliplatin-treated cancer cells, indicating a key role for elevated NKR-L expression in the immuno-sensitizing effects of oxaliplatin (Fig. 6H). These data highlight a previously unappreciated mechanism of the augmentation of anticancer immunity by chemotherapy, suggesting that the proper timing of chemotherapy and immunotherapy can help eliminate chemo-resistant and weakly immunogenic cancer cell variants (Fig. 6I).

## Discussion

Our data demonstrate that, in contrast to naïve CD8^+^ T cells which rely on CD28-mediated costimulation to initially respond to DC-presented antigens, effector CD8^+^ T cells rely on NKRs, especially DNAM-1, to costimulate responses to cancer cells which fail to express CD28 ligands. NKR-mediated costimulation is critically needed to reduce the TCR activation threshold of CTLs, allowing for effective CTL activation by cancer cells expressing low levels of cognate MHC I-peptide complexes. This NKR-delivered “alternative signal 2” is correlated with CTL function and survival in melanoma patients and can be manipulated to further enhance the TAA-specific CTL responsiveness for cancer therapy. These results advance our understanding of the ability of unmutated TAAs to serve as tumor-selective rejection antigens, and how CTLs selectively target cancer cells by recognizing the same unmutated “self” antigens on cancer cells but not healthy cells (**Extended Data Fig. 7**).

Lack of classical signal 2 delivery by cancer cells has been recognized in the field of CAR-T therapy as a factor limiting its efficacy. Synthetic CD28 signaling domains (or alternatively, 4–1BB or OX40) have been shown necessary to provide “artificial signal 2”, assuring persistence and therapeutic efficacy of modern CAR-T cell-based immunotherapies [4]. CAR-NK cells with CAR-linked DNAM-1 intracellular domain have been recently shown to have higher cytotoxic abilities vs. CAR-NK cells integrating CD28 signaling domain [30]. These observations, and our current results showing a particularly strong benefit of DNAM-1 costimulation in CTL responses against cancer cells expressing low levels of MHC I/TAA peptide complexes, provide a strong rationale to integrate DNAM-1 as a costimulatory component in the engineering of improved TCR-transgenic T cell and CAR-T cell products for therapeutic intervention.

The current designs of CAR-T cell constructs include intracellular domains providing signal 1 and 2 with same antigen/ligand binder. While such design enhances the potency of CARs, it does not enhance the discrimination between cancer and healthy cells expressing the same target antigens, introducing the risk of autoimmunity and T cell hyperactivation. In sharp contrast, our data demonstrate that the TAA-specific killing of cancer cells by CTLs can be achieved by double-transduction of CTLs with separate TCR and NKRs constructs, which bind to their separate ligands (pMHC I and NKR-Ls). This supports the feasibility of more selective “dual-recognition” systems involving the delivery of a) signal 1 by TCRs or CARs recognizing tumor antigens and b) delivery of signal 2 by modified NKRs or CARs recognizing NKR-Ls on cancer cells. Such “dual-recognition” may allow CTLs to selectively receive two separate cancer-specific signals to achieve higher functional avidity and enhanced recognition of cancer cells vs. healthy cells, even under conditions when both cell types expressing a comparable level of TAAs.

The heterogenous expression of multiple NKR-Ls, at baseline or in response to stressors such as chemo-, radio- or targeted therapies, suggest the general applicability of targeting NKR-Ls in the chemo/immunotherapy of diverse forms of solid cancer. While traditional approaches to combine chemo- and immunotherapy have focused on the induction of immunogenic cell death and depletion of Treg/MDSC by chemotherapy [31], our current data indicate potential synergy at the effector stage of anti-tumor T cell responses by sensitizing cancer cells to immune attack, with distinct implications for the relative timing of both therapeutic elements for optimal therapeutic impact.

Previous studies have also shown that therapeutic agents and irradiation which induce DNA damage responses can upregulate NKR-Ls in different types of cancers and enhance NK-mediated antitumor response [32, 33]. Paradoxically, high expression level of NKR-Ls has shown to be correlated with poor prognosis of cancer patients, which may result from the ability of soluble NKR-Ls released by cancer cells to block immune cell-expressed NKRs or induce their internalization [34–36]. These studies support the need for further in-depth analyses of the impact of different forms of chemo-, radio- and targeted therapy on the induction of cell surface versus soluble NKR-Ls in cancer cells.

Our current studies tested the role of NKR-mediated “alternative signal 2” during the priming stage of naïve CD8^+^ T cell activation and early effector phase of CTL activation. Our data raises the question of a role of NKRs at other activation stages of CD8^+^ T cells, including their exhaustion, and whether enhanced delivery of “alternative signal 2” may affect antitumor activity of other forms of cancer therapies such as immune checkpoint blockade. Our results raise the possibility that the NKR-mediated “alternative signal 2” may override the TCR-modulating suppressive signals provided by, such as CTLA4, PD1 or other inhibitory checkpoint molecules present on exhausted T cells and repetitively stimulated CAR T cells [37], potentially aiding them to survive and retain effector polyfunctionality and prolong cytotoxic activity against large tumor masses. Moreover, the DNAM-1-TIGIT axis is analogous to CD28-CTLA4 axis, as DNAM-1 and TIGIT compete for PVR in providing stimulatory versus inhibitory signals to CTLs [38, 39]. DNAM-1-negative CD8^+^ T cells have been identified as a dysfunctional T cell subpopulation which accumulates in the tumor-infiltrating lymphocyte population during disease progression [40, 41]. Meanwhile, the frequency of DNAM-1^high^CD8^+^ T cells appears to hold positive predictive value in patients receiving anti-TIGIT therapy [42]. These findings, together with our data showing that overexpression of NKRs benefits CTL function, suggest that by enhancing the bioavailability of PVR to DNAM-1, one may interfere with TIGIT-mediated suppression and potentially improve the efficacy of TIGIT blockade therapy.

In summary, we demonstrate that NKRs provide an important “alternative signal 2” at the effector stage of T cell activation, replacing CD28 signals which cannot be delivered by non-professional APCs, such as cancer cells. Our findings suggest that enhanced delivery of NKR-mediated “alternative signal 2” may be used to improve the antitumor activity of different forms of immunotherapy, ranging from adoptive T cell therapies to immune checkpoint blockade, and their combinations with other forms of cancer therapy, with the anticipation of improve patient outcomes. Prospective studies are planned to directly test these hypotheses.

## Methods

### Cell lines, media, and reagents

Human HLA-A*02:01^+^, MART-1^+^ melanoma cell lines Mel526 and Mel624 were the kind gifts of Dr. Steven Rosenberg (NCI; [15]). Human melanoma cell line 2183-Her4 was provided by Dr. Marc Ernstoff (Roswell Park). Human HLA-A*02:01^+^ cancer cell lines SW620 and Caco-2 (both colorectal cancer) and OVCAR3 (ovarian cancer) were purchased from ATCC. Mel526 and Mel624 were cultured in Dulbecco’s Modified Eagle Medium (Gibco) supplemented with 10% fetal bovine serum (FBS; Gibco). SW620 were cultured in Leibovitz’s L-15 medium (Gibco) supplemented with 10% FBS. Caco-2 were cultured in Minimum Essential Medium (Gibco) supplemented with 20% FBS. 2183-Her4 and OVCAR3 were cultured in RPMI1640 (Gibco) supplemented with 10% FBS. SW620 were incubated at 37°C without CO_2_. Other cells were incubated at 37°C with 5% CO_2_. CellGenix DC medium (Sartorius CellGenix) was used to generate monocyte-derived dendritic cells. AIM-V medium (Gibco) supplemented with 5% human serum (GeminiBio) was used as the base T cell culture medium. The following cytokines and reagents were used to generate immature DCs and induce DC maturation, or to activate and culture T cells: GM-CSF (Leukine sargramostim) was purchased from Partner Therapeutics; IL-4, IL-1β, TNFα, and IFN-γ were purchased from Miltenyi; IL-6 was purchased from R&D Systems; IFN-α (Intron A- IFN-α-2b) and IL-2 (Proleukin aldesleukin) was purchased from McKesson; PGE2 and poly-I:C were purchased from Sigma-Aldrich; IL-7 and IL-12p70 were purchased from Peprotech; Staphylococcus Enterotoxin B (SEB) was purchased from List Labs; Dynabeads Human T-Activator CD3/CD28 was purchased from Gibco; Purified Streptavidin was purchased from Biolegend to crosslink biotinylated-antibodies for short-term CTL stimulation. Oxaliplatin and *cis*-Diammineplatinum(II) Dichloride (Cisplatin) were purchased from Sigma-Aldrich. Information for antibodies used in this study is provided in Supplementary Tables 1–3.

### Human samples

Peripheral blood samples from deidentified healthy donors were obtained from the Roswell Park Donor Center. HLA-A*02:01^+^ peripheral blood leukapheresis packs were purchased from StemCell Technologies Inc, Vancouver, Canada.

### Generation of DCs

Peripheral blood mononuclear cells (PBMCs) were obtained from leukapheresis products using gradient centrifugation with Ficoll Paque Plus (Sigma-Aldrich). Fractions of monocytes and lymphocytes were further separated using density gradients made with Percoll (Sigma-Aldrich). Monocytes were purified by plastic adherence and cultured for 6 days in 24-well plates in CellGenix DC medium supplemented with 1000 IU/ml GM-CSF and 1000 IU/ml IL-4. At day 6, DCs were exposed to the following combinations of stimuli for 18 hours to induce standard mature DCs (sDCs; induced by 25 ng/ml IL-1β, 50 ng/ml TNFα, 1000 IU/ml IL-6, and 1 μM PGE_2;_ used in SEB-based polyclonal models of T cell activation); or α-type-1 polarized DCs (αDC1s; induced by 25 ng/ml IL-1β, 50 ng/ml TNFα, 3000 IU/ml IFN-α, 1000 IU/ml IFN-γ, and 20 μg/ml poly-I:C; used for the induction of MART-1-specific CTLs), as previously described [43, 44].

### DC induction of MART-1-specific CTLs in *in vitro* sensitization

To induce MART-1-specific CTLs, bulk CD8^+^ T cells were isolated from the lymphocyte fraction of PBMCs from HLA-A*02:01^+^ donors by magnetic cell separation using CD8 MicroBeads (Miltenyi). T cells were cocultured with 1 μg/ml 27L MART-1 (ELAGIGILTV; AnaSpec)-loaded autologous αDC1s in a 10:1 (T: DC) ratio. The cultures were supplemented with 50 IU/ml IL-2 and 10 ng/ml IL-7 at day 3 and every 2–3 days afterwards. MART-1-specific CTLs were sorted by flow cytometry based on the staining of dextramer (Immudex) at day 7. Sorted CTLs were incubated for at least 2 days before functional assays. MART-1-specific clone (clone 40) was established from the expanded metastatic tumor infiltrated lymph node cells of a melanoma cancer patient at the University of Lausanne, as described [17], and was restimulated *in vitro* using MART-1 peptide-loaded αDC1s using the same method.

### DC-induced polyclonal T cell activation and restimulation

To induce polyclonal activation of naïve CD8^+^ T cells by DCs, naïve CD8^+^ T cells were isolated, labeled with CFSE (Invitrogen), and cocultured with SEB (1 ng/ml)-loaded sDCs (generated from autologous monocytes) in a 5:1 (T: DC) ratio. When indicated, prior to coculture, T cells were incubated with anti-human NKG2D (clone 1D11) or anti-human DNAM-1 (clone DX11) (both 10 μg/ml) and DCs were incubated with recombinant CTLA-4-Ig (50 μg/ml) for 15 minutes at 37°C, to block NKG2D, DNAM-1, and B7 molecules, respectively. The cultures were supplemented with medium containing the same blocking antibodies along with 50 IU/ml IL-2 and 10 ng/ml IL-7 at day 2. T cell proliferation was analyzed based on the CFSE dilution using flow cytometry at day 5. When testing the restimulation of CTLs by DCs, Dynabeads-induced CTLs were cocultured with SEB (0.1 ng/ml) loaded autologous sDCs in a 5:1 (T: DC) ratio for 6 hours. When indicated, blocking Abs were applied using the same method as described above. Brefeldin A (Invitrogen) was added to the coculture 4 hours before the end of incubation. CTL restimulation was analyzed based on intracellular IFN-γ staining using flow cytometry.

### CTL induction by Dynabeads

Naïve CD8^+^ T cells were isolated from PBMCs or the lymphocyte fraction of PBMCs by magnetic cell separation using EasySep^™^ Human Naïve CD8^+^ T Cell Isolation Kit II (StemCell). Cells were then activated at 8×10^4^ cells per well in 96-well round-bottomed plates with an equivalent number of washed CD3/CD28-coated Dynabeads, 50 IU/ml IL-2, 10 ng/ml IL-7, and 10 ng/ml IL-12 in 200 μl of AIM-V medium supplemented with 5% human serum (starting point = day 0). Cells were activated for 48 hours, then the beads were magnetically removed, and the cells were incubated in 24-well plates with 50 IU/ml IL-2 and 10 ng/ml IL-7. Cultures were split and replenished with fresh medium supplemented with IL-2 and IL-7 every 2–3 days. Effector CTLs were harvested for functional assays between days 7 and 14.

### T cell activation by immobilized antibodies

To activate naïve CD8^+^ T cells, 1μg/ml solution of anti-human CD3 (clone OKT3) was prepared in sterile PBS and dispensed to 96-well flat bottom plate. The plate was sealed and incubated at 4 °C overnight. When indicated, OKT3-coated plate was washed and coated with the following antibody solutions at 37 °C for 2 hours: 10 μg/ml mouse IgG1, κ isotype control (clone MOPC-21), 10 μg/ml anti-human NKG2D (clone 1D11), or 10 μg/ml anti-human DNAM-1 (clone DX11). CFSE-labeled naïve CD8^+^ T cells were aliquoted into microwells of antibody-coated plate at 2×10^4^ cells per well in 200 μl of culture medium supplemented with 50 IU/ml IL-2 and 10 ng/ml IL-7. To activate CD28, anti-human CD28 (clone CD28.2) was added into cell suspension at 5 μg/ml. On day 3, for each condition, 100 μl supernatant was carefully harvested for IFN-γ ELISA and replenished by 100 μl fresh medium supplemented with IL-2 and IL-7. T cell proliferation was analyzed based on the CFSE dilution using flow cytometry at day 6. To stimulate effector CTLs, OKT3 was coated at different concentrations as indicated in figure legends, and other antibody stimuli were given in the same method as described above. After 4 hours of stimulation, supernatant was harvested for IFN-γ ELISA, and cells were harvested for cytokine and gene expression profiling. For examining CTL activation by intracellular IFN-γ, brefeldin A was added after 2 hours of stimulation and cells were cultured for additional 4 hours before intracellular staining.

### T cell transduction

The retroviral vector production and transduction of NY-ESO-1-specific TCR genes 19305DP and CD8SP were performed as previously described [21]. RetroNectin (catalog #T1008; TaKaRa) was used for coating of retrovirus. Transduction efficiency was determined by NY-ESO-1_157–165_ (SLLMWITQC) tetramer (iTAg MHC tetramer; MBL International). To generate NKG2D/DNAM-1 co-expressing vector, human full length CD314 and CD226 coding sequences were fused via P2A-skipping site and cloned into the previously described MSCV-based retroviral vector [45]. To generate TCR/NKRs double-transgenic T cells, HLA-A*02:01^+^ PBMCs were preactivated using 50 ng/ml OKT3 and 300 IU/ml IL-2 (starting point = day 0). On day 2, cells were added to the 19305DP retrovirus-coated plate with T cell culture medium containing 300 IU/ml IL-2, spined 1000×g at 32 °C for 10 minutes and incubated at 37 °C with 5% CO2. The same process was repeated after 8 hours of incubation. On day 3, cells were transduced with NKG2D/DNAM-1 co-expressing retrovirus using the same protocol. On day 4, double-transgenic T cells were harvested and cultured in T cell culture medium containing 300 IU/ml IL-2. CD8^+^tetramer^+^ T cells were flow sorted on day 7 and cultured for at least 2 days in T cell culture medium containing 50 IU/ml IL-2 and 10 ng/ml IL-7 before functional assays.

### Loading cancer cells with tumor-associated antigens (TAAs)

To model cancer cells expressing different levels of TAAs as target cells for TAA-specific CTL recognition and killing, HLA-A*02:01^+^ cancer cells (SW620 and Caco-2) were suspended at 1×10^6^ cells/ml in T cell culture medium mixed with 27L MART-1 (ELAGIGILTV; AnaSpec) or NY-ESO-1 (SLLMWITQC; MBL International) at indicated concentrations. Cell suspensions were incubated in 37 °C incubator for 2 hours with mixing by vortex every 30 minutes, followed by 3 times washing using T cell culture medium to remove residual free peptides. When using wild type MART-1 (EAAGIGILTV; AnaSpec), peptides were directly added into the coculture of cancer cells and CTLs at indicated concentrations without incubation and washing steps.

### Antibody blockade of the CTL-cancer cell interactions

To block target molecules, prior of coculture, CTLs and cancer cells were incubated with indicated antibodies for 15 minutes at 37 °C: For CTLs, anti-human NKG2D (clone 1D11), anti-human DNAM-1 (clone DX11), anti-human CD2 (clone TS1/8) were used at 10 μg/ml. Mouse IgG1, κ (clone MOPC-21) was used as the isotype control for anti-NKG2D and DNAM-1. For cancer cells, anti-human HLA-ABC (clone W6/32) were used at 5 μg/ml as a suboptimal concentration to partially block MHC class I.

### IFN-γ ELISA

IFN-γ in supernatant of T cell culture was measured using a human IFN-γ ELISA kit according to the manufacturer’s protocol (catalog #DY285B, R&D Systems). Plate washing was performed using BioTek 405LS Microplate Washer. Plate reading was performed using BioTek Epoch Microplate Spectrophotometer with Gen5 software.

### IFN-γ ELISpot assay

To perform IFN-γ ELISpot assays, 96-well MultiScreen Filter Plates (Millipore) were coated with 10 μg/ml anti-human IFN-γ mAb 1-D1K (Mabtech) at 4 °C overnight, washed with PBS, and blocked with T cell culture medium at 37 °C for 1 hour before use. 2×10^4^ cells/well TAA loaded cancer cells or melanoma cancer cells were cocultured with effector cells (numbers were indicated in figure legends) in 100 μl T cell culture medium at 37°C with 5% CO_2_ for 24 hours. Plates were rinsed with PBS containing 0.05% Tween-20 and coated with 10 μg/ml anti-human IFN-γ mAb 7-B6–1-Biotin (Mabtech) at 4 °C overnight. Immunospots were developed using VECTASTAIN Elite ABC-HRP Kit, Peroxidase (Standard) and AEC Substrate Kit, Peroxidase (HRP), (3-amino-9-ethylcarbazole) according to the manufacturer’s protocol (catalog #PK-6100 and SK4200, Vector Laboratories, Inc.). Spots were imaged and enumerated using CTL ImmunoSpot S6 Core Analyzer (Cellular Technology Ltd).

### Cytotoxicity assays

For LDH cytotoxicity assay, 1×10^4^ MART-1-specific CTLs were preincubated with indicated blocking antibodies as described above and cocultured with 2×10^4^ MART-1-loaded SW620 in 200 μl T cell culture medium in 96-well plate at 37 °C with 5% CO_2_ for 24 hours. LDH activity of each sample was determined using CyQUANT LDH Cytotoxicity Assay Kit according to the manufacturer’s protocol (catalog #C20300, Thermo Fisher Scientific). For apoptosis assay, 1×10^5^ T cells were cocultured with 2×10^5^ peptide-loaded SW620 in 500 μl T cell culture medium in 24-well ultra-low attachment plate at 37 °C with 5% CO_2_ for 24 hours. Cells were then harvested and stained using Alexa Fluor 488 annexin V/Dead Cell Apoptosis Kit (catalog #V13245, Invitrogen) with an adapted protocol. In brief, cells were washed in cold PBS, and resuspended in 100 μl 1× annexin-binding buffer containing Alexa Fluor 488 annexin V and BV786 mouse anti-human CD8 (clone RPA-T8). Samples were incubated at room temperature for 15 minutes and 400 μl 1× annexin-binding buffer containing 1 μM DAPI (Sigma-Aldrich) were added. Samples were then kept on ice and acquired on flow cytometer immediately.

### Intracellular Ca^2+^ flux assay

Cell labeling, stimulation and detection were performed in HBSS (1X) with calcium chloride, magnesium chloride (Gibco) supplemented with 2% FBS. CTLs were labeled with Fluo-4, AM using Fluo-4 Calcium Imaging Kit according to the manufacturer’s protocol (catalog #F10489, Thermo Fisher Scientific). Cells were then incubated with the following antibodies either alone or in combination at 37 °C for 15 minutes: biotin anti-human CD3 (clone OKT3), biotin anti-human NKG2D (clone 1D11), biotin anti-human DNAM-1 (clone 11A8), biotin anti-human CD28 (clone CD28.2). After incubation, cells were collected by centrifugation and resuspended in assay buffer containing 1 μM DAPI. Intracellular Ca^2+^ levels over time were detected by flow cytometry. For each sample, baseline fluorescent signal was recorded for 30 seconds, then streptavidin was added at a final concentration of 20 μg/ml for crosslinking and the fluorescent signal was followed for additional 9 minutes.

### Flow cytometry

Surface staining was performed in PBS containing 2% BSA, 1 mM EDTA and 0.02% NaN_3_. For IFN-γ intracellular staining, T cells were stimulated in the presence of brefeldin A as described above. Cells were fixed and permeabilized using BD Fixation/Permeabilization Kit according to the manufacturer’s protocol (catalog #554714, BD Biosciences), and stained with APC mouse anti-human IFN-γ (clone B27). For degranulation assay, 2×10^5^ MART-1-specific CTLs were cocultured with 8×10^5^ MART-1-loaded SW620 in T cell culture medium for 6 hours at 37 °C, in the presence of PE mouse anti-human CD107a (clone H4A3) and BD GolgiStop protein transport inhibitor (containing monensin) (catalog #554724, BD Biosciences). Cells were then stained with BV786 mouse anti-human CD8 (clone RPA-T8) and resuspended in staining buffer containing 1 μM DAPI. For cell sorting, sample preparation was performed in PBS containing 2% BSA with 1% penicillin/ streptomycin (Gibco). To detect CTL-cancer cell conjugates, MART-1-specific CTLs were labelled with CM-Dil according to the manufacturer’s protocol (catalog #C7001, Invitrogen), incubated with indicated blocking antibodies, and cocultured with CFSE-labelled MART-1-loaded SW620 in 100 μl T cell culture medium at E:T ratio of 1:1. Samples were incubated at 37 °C incubator for 10 minutes, then resuspended by adding 400 μl PBS containing 2% BSA and mild pipetting, and immediately acquired on flow cytometer. Flow cytometry and cell sorting were performed using BD LSRFortessa Cell Analyzer and BD FACSAria II Cell Sorter (BD Biosciences). Data was analyzed using FlowJo software (FlowJo, LLC). Detail information of antibodies for flow cytometry were listed in Supplementary Table 2.

### Analysis of synapse formation by ImageStream

DC-sensitized MART-1-specific CTLs were incubated with CFSE-labelled MART-1-loaded SW620 at E:T ratio of 1:1 in 37°C incubator for 15 minutes. Samples were fixed with 4% Paraformaldehyde and permeabilized using 0.3% Triton X-100, both at room temperature for 10 minutes. Samples were stained with PE anti-human CD3, PerCP/Cyanine5.5 anti-human CD11a/CD18, BV510 anti-human NKG2D or BV510 anti-human DNAM-1, and Alexa Fluor 647 Phalloidin at room temperature for 30 minutes. After staining, samples were washed and immediately acquired on ImageStream^X^ MKII (Amnis). Data analysis was performed using IDEAS software (Amnis). Detail information of antibodies for ImageStream were listed in Supplementary Table 2.

### Western blot

To prepare samples for western blot, 3×10^6^ CTLs were incubated with 0.5 μg/ml OKT3 with or without 10 μg/ml DNAM-1 in T cell culture medium at 37°C for 15 minutes. After incubation, cells were collected by centrifugation and resuspended in 200 μl T cell culture medium. Samples were mixed with 200 μl HBSS (with CaCl_2_, MgCl_2_) containing 40 μg/ml streptavidin and incubated at 37°C water bath for indicated time length. Cells were collected by centrifugation at 4 °C 1250×g for 2 minutes and washed by ice-cold PBS. Proteins were extracted by lysis buffer containing 1× HALT Protease and Phosphatase Inhibitor Cocktail and quantitated using Pierce BCA Protein Assay Kit, with both reagents purchased from Thermo Fisher Scientific. SDS PAGE was performed using 4–15% Mini-PROTEAN TGX Precast Protein Gels with Precision Plus Protein Dual Color Standards. Wet transfer was performed using PVDF Membrane. All were purchased from Bio-Rad Life Science. After protein transfer, membranes were blocked with 5% BSA for 30 minutes at room temperature and then incubated with primary antibodies at 4°C overnight. Membranes were then washed and incubated with fluorescent-conjugated secondary antibodies for 1 hour at room temperature. The protein bands were imaged using Odyssey Fc Imager with Image Studio Lite software (LI-COR Biosciences). Detail information of antibodies for western blot were listed in Supplementary Table 3.

### Single cell multiplex cytokine profiling

CTLs were stimulated by immobilized antibodies for 4 hours as described above. Cells were harvested for cytokine profiling using Single-Cell Adaptive Immune Chip and Panel according to the manufacturer’s protocol (catalog #ISOCODE-1001-4 and PANEL-1001-4; IsoPlexis). In brief, cells were stained with Stain Cell Membrane 405 (catalog #STAIN-1001-1; IsoPlexis) and approximately 30,000 cells were loaded onto the chip containing 12,000 chambers prepatterned with an array of 32 cytokine capture antibodies. Chips were incubated in the IsoLight system at 37 °C with 5% CO_2_ for an additional 16 hours and labelled with detection antibodies. The fluorescent signals of each cytokine at single-cell level were detected and analyzed by IsoLight system with IsoSpeak software (IsoPlexis).

### RNA sequencing

Dynabead-induced CTLs from 3 donors were stimulated by anti-human CD3 (OKT3) with or without anti-human DNAM-1 (DX11) for 4 hours. Total RNA was prepared using RNeasy Mini Kit and RNase-Free DNase Set according to the manufacturer’s protocol (catalog #74104 and 79254; Qiagen). Paired-end sequencing was performed by the Roswell Park Genomics Shared Resource on an Illumina NovaSeq 6000. Reads were aligned to the human genome (GRCh38) using STAR (version 2.7.9a) and transcripts were quantified by FeatureCounts from the Subread package (version 2.0.1) [46, 47]. Normalization and differential expression analysis was completed with DESeq2 while modeling sample donor as a covariate [48]. Gene Set Enrichment Analysis (GSEA) was performed with the fgsea R package (version 1.18.0) using rank ordered differential expression and gene sets derived from the Molecular Signatures Database (MSigDB) [49].

### qPCR

Cancer cells were treated with indicated concentrations of chemotherapeutic drugs for 24 hours. RNA was isolated as described above. Reverse transcription was performed using qScript cDNA Synthesis Kit (QuantaBio) according to the manufacturer’s protocol with T100 Thermal Cycler (Bio-Rad). Real-Time PCR assay was performed using iTaq Universal Probes Supermix and CFX96 Real-Time PCR System (both from Bio-Rad). HPRT1 was used as endogenous control (catalog #4325801; Life technologies). Primer information was listed in Supplementary Table 4.

### Analysis of NKR expression from the DICE project

Gene expression data were retrieved from the DICE (Database of Immune Cell Expression, Expression quantitative trait loci (eQTLs) and Epigenomics) project (https://dice-database.org/). Mean TPM of indicated NKR genes were compared between different cell types.

### Analysis of TCGA-SKCM metastatic melanoma cancer cohort

TCGA-SKCM, COAD and OV expression and clinical annotations were obtained from the Genomic Data Commons data portal and processed via TCGAbiolinks package in R using TCGAWorkflow guided practices [50]. For SKCM, only metastatic samples were utilized in subsequent analyses. Associations between normalized *DNAM-1 (CD226)* and *NKG2D (KLRK1)* expression and typical CD8^+^ T Cell *(CD3G, CD8A, CD8B)* and NK cell *(NCR1, NCR2, NCAM1)* lineage and functional *(IFNG, GZMK, GZMB)* markers was performed via Spearman correlation analysis. Overall survival analysis was conducted by Kaplan-Meier curve and log-rank test using the survival package in R. High and low subsets of indicated genes were defined using median expression or scaled z-scores. Hazard ratios for overall survival were calculated using individual log-transformed gene expression. A ‘functional hotness score’ for combined expression of *CD8A, DNAM-1,* and *NKG2D* was calculated as previously described [51].

### Statistical analysis

All statistical analyses were performed using GraphPad Prism 9. Data from replicate cultures were presented as mean ± SD. Data from multiple donors were presented as mean ± SEM. The numbers of replicates and donors were provided in the figure legends. A Student’s *t*-test or paired *t*-test was used to compare two independent or matched groups. *P*-values < 0.05 were considered to be significant (*P < 0.05, **P < 0.01, ***P < 0.001).

## References

[1] Bretscher P. and Cohn M., A theory of self-nonself discrimination. Science, 1970. 169(3950): p. 1042–9. 4194660 10.1126/science.169.3950.1042

[2] Esensten J.H., , CD28 Costimulation: From Mechanism to Therapy. Immunity, 2016. 44(5): p. 973–88. 27192564 10.1016/j.immuni.2016.04.020 PMC4932896

[3] Acuto O. and Michel F., CD28-mediated co-stimulation: a quantitative support for TCR signalling. Nat Rev Immunol, 2003. 3(12): p. 939–51. 14647476 10.1038/nri1248

[4] Jackson H.J., Rafiq S., and Brentjens R.J., Driving CAR T-cells forward. Nat Rev Clin Oncol, 2016. 13(6): p. 370–83. 27000958 10.1038/nrclinonc.2016.36 PMC5529102

[5] Raulet D.H., , Regulation of ligands for the NKG2D activating receptor. Annu Rev Immunol, 2013. 31: p. 413–41. 23298206 10.1146/annurev-immunol-032712-095951 PMC4244079

[6] Martinet L. and Smyth M.J., Balancing natural killer cell activation through paired receptors. Nat Rev Immunol, 2015. 15(4): p. 243–54. 25743219 10.1038/nri3799

[7] Bauer S., , Activation of NK cells and T cells by NKG2D, a receptor for stress-inducible MICA. Science, 1999. 285(5428): p. 727–9. 10426993 10.1126/science.285.5428.727

[8] Groh V., , Costimulation of CD8alphabeta T cells by NKG2D via engagement by MIC induced on virus-infected cells. Nat Immunol, 2001. 2(3): p. 255–60. 11224526 10.1038/85321

[9] Pievani A., , Dual-functional capability of CD3+CD56+ CIK cells, a T-cell subset that acquires NK function and retains TCR-mediated specific cytotoxicity. Blood, 2011. 118(12): p. 3301–10. 21821703 10.1182/blood-2011-02-336321

[10] Shibuya A., , DNAM-1, a novel adhesion molecule involved in the cytolytic function of T lymphocytes. Immunity, 1996. 4(6): p. 573–81. 8673704 10.1016/s1074-7613(00)70060-4

[11] Meresse B., , Coordinated induction by IL15 of a TCR-independent NKG2D signaling pathway converts CTL into lymphokine-activated killer cells in celiac disease. Immunity, 2004. 21(3): p. 357–66. 15357947 10.1016/j.immuni.2004.06.020

[12] Verneris M.R., , Role of NKG2D signaling in the cytotoxicity of activated and expanded CD8+ T cells. Blood, 2004. 103(8): p. 3065–72. 15070686 10.1182/blood-2003-06-2125

[13] Horng T., Bezbradica J.S., and Medzhitov R., NKG2D signaling is coupled to the interleukin 15 receptor signaling pathway. Nat Immunol, 2007. 8(12): p. 1345–52. 17952078 10.1038/ni1524

[14] Coulie P.G., , A new gene coding for a differentiation antigen recognized by autologous cytolytic T lymphocytes on HLA-A2 melanomas. J Exp Med, 1994. 180(1): p. 35–42. 8006593 10.1084/jem.180.1.35 PMC2191574

[15] Kawakami Y., , Cloning of the gene coding for a shared human melanoma antigen recognized by autologous T cells infiltrating into tumor. Proc Natl Acad Sci U S A, 1994. 91(9): p. 3515–9. 8170938 10.1073/pnas.91.9.3515 PMC43610

[16] Pittet M.J., , High frequencies of naive Melan-A/MART-1-specific CD8(+) T cells in a large proportion of human histocompatibility leukocyte antigen (HLA)-A2 individuals. J Exp Med, 1999. 190(5): p. 705–15. 10477554 10.1084/jem.190.5.705 PMC2195613

[17] Dutoit V., , Functional avidity of tumor antigen-specific CTL recognition directly correlates with the stability of MHC/peptide multimer binding to TCR. J Immunol, 2002. 168(3): p. 1167–71. 11801651 10.4049/jimmunol.168.3.1167

[18] Garrido F., , The urgent need to recover MHC class I in cancers for effective immunotherapy. Curr Opin Immunol, 2016. 39: p. 44–51. 26796069 10.1016/j.coi.2015.12.007 PMC5138279

[19] Abdul Razak F.R., , CD58 mutations are common in Hodgkin lymphoma cell lines and loss of CD58 expression in tumor cells occurs in Hodgkin lymphoma patients who relapse. Genes Immun, 2016. 17(6): p. 363–6. 27467287 10.1038/gene.2016.30

[20] Grasso C.S. and Giannakis M., Genomic mechanisms of immune evasion in colorectal cancer: from discovery to clinical practice. Oncotarget, 2018. 9(73): p. 33743–33744. 30333906 10.18632/oncotarget.26105 PMC6173469

[21] Matsuzaki J., , A rare population of tumor antigen-specific CD4(+)CD8(+) double-positive alphabeta T lymphocytes uniquely provide CD8-independent TCR genes for engineering therapeutic T cells. J Immunother Cancer, 2019. 7(1): p. 7. 30626427 10.1186/s40425-018-0467-y PMC6325755

[22] Rossi J., , Preinfusion polyfunctional anti-CD19 chimeric antigen receptor T cells are associated with clinical outcomes in NHL. Blood, 2018. 132(8): p. 804–814. 29895668 10.1182/blood-2018-01-828343 PMC6107882

[23] Creelan B.C., , Tumor-infiltrating lymphocyte treatment for anti-PD-1-resistant metastatic lung cancer: a phase 1 trial. Nat Med, 2021. 27(8): p. 1410–1418. 34385708 10.1038/s41591-021-01462-y PMC8509078

[24] Abbas H.A., , Single-cell polyfunctional proteomics of CD4 cells from patients with AML predicts responses to anti-PD-1-based therapy. Blood Adv, 2021. 5(22): p. 4569–4574. 34555853 10.1182/bloodadvances.2021004583 PMC8759127

[25] Upshaw J.L., , NKG2D-mediated signaling requires a DAP10-bound Grb2-Vav1 intermediate and phosphatidylinositol-3-kinase in human natural killer cells. Nat Immunol, 2006. 7(5): p. 524–32. 16582911 10.1038/ni1325

[26] Prajapati K., , Functions of NKG2D in CD8(+) T cells: an opportunity for immunotherapy. Cell Mol Immunol, 2018. 15(5): p. 470–479. 29400704 10.1038/cmi.2017.161 PMC6068164

[27] Zhang Z., , DNAM-1 controls NK cell activation via an ITT-like motif. J Exp Med, 2015. 212(12): p. 2165–82. 26552706 10.1084/jem.20150792 PMC4647266

[28] Best J.A., , Transcriptional insights into the CD8(+) T cell response to infection and memory T cell formation. Nat Immunol, 2013. 14(4): p. 404–12. 23396170 10.1038/ni.2536 PMC3689652

[29] Cerboni C., , The DNA Damage Response: A Common Pathway in the Regulation of NKG2D and DNAM-1 Ligand Expression in Normal, Infected, and Cancer Cells. Front Immunol, 2014. 4: p. 508. 24432022 10.3389/fimmu.2013.00508 PMC3882864

[30] Huang Y., , DNAM1 and 2B4 Costimulatory Domains Enhance the Cytotoxicity of Anti-GPC3 Chimeric Antigen Receptor-Modified Natural Killer Cells Against Hepatocellular Cancer Cells in vitro. Cancer Manag Res, 2020. 12: p. 3247–3255. 32440221 10.2147/CMAR.S253565 PMC7217313

[31] Salas-Benito D., , Paradigms on Immunotherapy Combinations with Chemotherapy. Cancer Discov, 2021. 11(6): p. 1353–1367. 33712487 10.1158/2159-8290.CD-20-1312

[32] Soriani A., , ATM-ATR-dependent up-regulation of DNAM-1 and NKG2D ligands on multiple myeloma cells by therapeutic agents results in enhanced NK-cell susceptibility and is associated with a senescent phenotype. Blood, 2009. 113(15): p. 3503–11. 19098271 10.1182/blood-2008-08-173914

[33] Weiss T., , NKG2D-Dependent Antitumor Effects of Chemotherapy and Radiotherapy against Glioblastoma. Clin Cancer Res, 2018. 24(4): p. 882–895. 29162646 10.1158/1078-0432.CCR-17-1766

[34] Groh V., , Tumour-derived soluble MIC ligands impair expression of NKG2D and T-cell activation. Nature, 2002. 419(6908): p. 734–8. 12384702 10.1038/nature01112

[35] Li K., , Clinical significance of the NKG2D ligands, MICA/B and ULBP2 in ovarian cancer: high expression of ULBP2 is an indicator of poor prognosis. Cancer Immunol Immunother, 2009. 58(5): p. 641–52. 18791713 10.1007/s00262-008-0585-3 PMC11030581

[36] Paschen A., , Differential clinical significance of individual NKG2D ligands in melanoma: soluble ULBP2 as an indicator of poor prognosis superior to S100B. Clin Cancer Res, 2009. 15(16): p. 5208–15. 19671853 10.1158/1078-0432.CCR-09-0886

[37] Good C.R., , An NK-like CAR T cell transition in CAR T cell dysfunction. Cell, 2021. 184(25): p. 6081–6100 e26. 34861191 10.1016/j.cell.2021.11.016 PMC8827167

[38] Alteber Z., , Therapeutic Targeting of Checkpoint Receptors within the DNAM1 Axis. Cancer Discov, 2021. 11(5): p. 1040–1051. 33687987 10.1158/2159-8290.CD-20-1248

[39] Chiang E.Y. and Mellman I., TIGIT-CD226-PVR axis: advancing immune checkpoint blockade for cancer immunotherapy. J Immunother Cancer, 2022. 10(4). 10.1136/jitc-2022-004711 PMC8981293 35379739

[40] Weulersse M., , Eomes-Dependent Loss of the Co-activating Receptor CD226 Restrains CD8(+) T Cell Anti-tumor Functions and Limits the Efficacy of Cancer Immunotherapy. Immunity, 2020. 53(4): p. 824–839 e10. 33053331 10.1016/j.immuni.2020.09.006

[41] Braun M., , CD155 on Tumor Cells Drives Resistance to Immunotherapy by Inducing the Degradation of the Activating Receptor CD226 in CD8(+) T Cells. Immunity, 2020. 53(4): p. 805–823 e15. 33053330 10.1016/j.immuni.2020.09.010

[42] Jin H.S., , CD226(hi)CD8(+) T Cells Are a Prerequisite for Anti-TIGIT Immunotherapy. Cancer Immunol Res, 2020. 8(7): p. 912–925. 32265229 10.1158/2326-6066.CIR-19-0877

[43] Mailliard R.B., , alpha-type-1 polarized dendritic cells: a novel immunization tool with optimized CTL-inducing activity. Cancer Res, 2004. 64(17): p. 5934–7. 15342370 10.1158/0008-5472.CAN-04-1261

[44] Watchmaker P.B., , Independent regulation of chemokine responsiveness and cytolytic function versus CD8+ T cell expansion by dendritic cells. J Immunol, 2010. 184(2): p. 591–7. 20018619 10.4049/jimmunol.0902062 PMC2922038

[45] Tsuji T., , Rapid Construction of Antitumor T-cell Receptor Vectors from Frozen Tumors for Engineered T-cell Therapy. Cancer Immunol Res, 2018. 6(5): p. 594–604. 29588318 10.1158/2326-6066.CIR-17-0434 PMC5932240

[46] Dobin A., , STAR: ultrafast universal RNA-seq aligner. Bioinformatics, 2013. 29(1): p. 15–21. 23104886 10.1093/bioinformatics/bts635 PMC3530905

[47] Liao Y., Smyth G.K., and Shi W., featureCounts: an efficient general purpose program for assigning sequence reads to genomic features. Bioinformatics, 2014. 30(7): p. 923–30. 24227677 10.1093/bioinformatics/btt656

[48] Love M.I., Huber W., and Anders S., Moderated estimation of fold change and dispersion for RNA-seq data with DESeq2. Genome Biol, 2014. 15(12): p. 550. 25516281 10.1186/s13059-014-0550-8 PMC4302049

[49] Korotkevich G., , Fast gene set enrichment analysis. BioRxiv, 2016: p. 060012.

[50] Mounir M., , New functionalities in the TCGAbiolinks package for the study and integration of cancer data from GDC and GTEx. PLoS Comput Biol, 2019. 15(3): p. e1006701. 30835723 10.1371/journal.pcbi.1006701 PMC6420023

[51] Katsuta E., , Cytotoxic T-lymphocyte infiltration and chemokine predict long-term patient survival independently of tumor mutational burden in triple-negative breast cancer. Ther Adv Med Oncol, 2021. 13: p. 17588359211006680. 33868461 10.1177/17588359211006680 PMC8024454

